# Effects of different doses of dexamethasone as local anesthetic adjuvant on brachial plexus block: A protocol for systematic review and meta analysis

**DOI:** 10.1097/MD.0000000000025651

**Published:** 2021-04-30

**Authors:** Shuai Zhang, Meiyan Song, Wei An, Zhongyi Wang

**Affiliations:** Department of Anesthesiology, Harrison International Peace Hospital, Hebei Medical University, Hengshui, Hebei Province, China.

**Keywords:** brachial plexus block, dexamethasone, meta-analysis, protocol

## Abstract

**Background:**

Dexamethasone has been widely used in brachial plexus block to enhance the effects of brachial plexus block. However, the clinical findings are not consistent with the dosage of dexamethasone prolonging local anesthetic nerve block. Therefore, the purpose of this study was to explore the effects of different doses of dexamethasone as local anesthetic adjuvant on brachial plexus block through network meta-analysis.

**Methods:**

We searched PubMed, Web of Science, Cochrane Library, and Embase databases to collect all randomized controlled trials (RCTs) of different doses of dexamethasone as local anesthetic adjuvant on brachial plexus block until March 2021. Two researchers then independently screened articles, extracted data, and evaluated the quality of selected literatures. All data was processed by Stata 14.0 and WinBUGS 1.4.3.software.

**Results:**

The results of this meta-analysis will be submitted to a peer-reviewed journal for publication.

**Conclusion:**

Our study is expected to provide high-quality evidence-based medicine advice for the effects of different doses of dexamethasone as local anesthetic adjuvant on brachial plexus block.

**Ethics and dissemination:**

Ethical approval was not required for this study. The systematic review will be published in a peer-reviewed journal, presented at conferences, and shared on social media platforms.

**OSF REGISTRATION NUMBER:**

DOI 10.17605/OSF.IO/PZ5WR.

## 1 Introduction

In upper limb surgery, compared with general anesthesia, brachial plexus block has various advantages, including low cost, less adverse reactions, less postoperative pain, and short hospital stay, which can provide not only intraoperative analgesia, but also postoperative analgesia.^[1–3]^ However, the action time of local anesthetics is limited, so it cannot provide postoperative analgesia.^[4]^ Postoperative pain can easily increase the stress response to body, increase the stress response to substances in blood vessels, cause vasospasm, and release many damage factors such as catecholamine and prostaglandins through neurohumoral factors. Strong contraction of small blood vessels could induce vascular crisis. Therefore, postoperative analgesia is particularly important.^[5]^

Dexamethasone is a local anesthetic adjuvant and widely used to enhance the effects of brachial plexus block.^[6]^ Studies have revealed that dexamethasone can prolong the time of local anesthetic nerve block analgesia.^[7–9]^ However, the results of clinical studies are not consistent with the dosage of dexamethasone prolonging local anesthetic nerve block. At present, it is recognized that 5 mg of dexamethasone is the highest dose of dexamethasone to prolong the blocking effects of local anesthetic.^[10–12]^ However, different studies have different conclusions on whether the blocking effects increase with the increase of the dose below 5 mg.^[10,12]^

The optimal dose of dexamethasone is still unclear. In the study of Chazapi et al, the dose of dexamethasone was 4 mg^1^ and the most common dose of brachial plexus block is 8 mg. For brachial plexus block, 4 mg of perinerve dexamethasone provided longer sensory, motor block, and analgesic effects than intravenous administration.^[13]^ On the other hand, 8 to 10 mg of dexamethasone could provide a similar duration of block locally or intravenously.^[14]^ Compared with the most commonly used dose of 8 mg of dexamethasone, a lower dose of dexamethasone may provide results similar to those of postoperative analgesia. In fact, a recent study has proved that lower doses (such as 1 mg) and higher doses of dexamethasone during analgesia can provide similar effects.^[6]^ This study will be divided into different doses of dexamethasone adopting the method of network Meta to compare, thus providing comprehensive and reliable evidence of evidence-based medicine for clinical practices.

## 2 Methods

### 2.1 Study registration

The protocol of this review was registered in OSF (OSF registration number: doi 10.17605/OSF.IO/PZ5WR). It was reported to follow the statement guidelines of preferred reporting items for systematic reviews and meta-analyses protocol.^[15]^

### 2.2 Inclusion criteria for study selection

#### 2.2.1 Types of studies

All randomized controlled trial (RCTs) of different doses of dexamethasone as local anesthetic adjuvant on brachial plexus block.

#### 2.2.2 Types of participants

Patients with brachial plexus block and patients older than 18 years old.

#### 2.2.3 Types of interventions

Different doses of dexamethasone were used as local anesthetic adjuvant to block brachial plexus nerve.

#### 2.2.4 Types of outcome measures

The main outcome index is the duration of analgesia, and is defined as the interval between the completion of local anesthetic injection and the first administration of analgesics. The secondary outcome index includes the duration of sensory block and motor block, and is defined as the interval between the completion of local anesthetic injection and the recovery of nerve block.

### 2.3 Exclusion criteria

1. Non-RCT.
2. Animal experiments, case reports and reviews, etc.
3. Repeatedly detected or published literature.
4. Being unable to obtain complete data or full text literature.

### 2.4 Data sources

PubMed, Web of Science, Cochrane Library, and EMBASE Database were systematically searched. The time for literature retrieval was set to build the database until March 2021.

### 2.5 Searching strategy

The details of PubMed's search strategies are illustrated in Table 1, including all search terms, while similar search strategies are applied to other electronic databases.

**Table 1:** Search strategy in PubMed database.

Number	Search terms
#1	Dexamethasone [MeSH]
#2	Hexadecadrol [Title/Abstract]
#3	Alcon Brand of Dexamethasone[Title/Abstract]
#4	Decaject[Title/Abstract]
#5	Decaject-L.A.[Title/Abstract]
#6	Decameth[Title/Abstract]
#7	Decaspray[Title/Abstract]
#8	Dexasone[Title/Abstract]
#9	Dexpak[Title/Abstract]
#10	ECR Brand of Dexamethasone[Title/Abstract]
#11	Foy Brand of Dexamethasone[Title/Abstract]
#12	Hexadrol[Title/Abstract]
#13	ICN Brand of Dexamethasone[Title/Abstract]
#14	Maxidex[Title/Abstract]
#15	Merck Brand of Dexamethasone[Title/Abstract]
#16	Merz Brand 1 of Dexamethasone[Title/Abstract]
#17	Merz Brand 2 of Dexamethasone[Title/Abstract]
#18	Methylfluorprednisolone[Title/Abstract]
#19	Millicorten[Title/Abstract]
#20	Oradexon[Title/Abstract]
#21	Decaject L.A.[Title/Abstract]
#22	or/1-21
#23	Brachial plexus block[Title/Abstract]
#24	Nerve block[Title/Abstract]
#25	Brachial plexus nerve block[Title/Abstract]
#26	Branchial nerve block[Title/Abstract]
#27	or/23-26
#28	Random∗[Title/Abstract]
#29	#22 and #27 and #28

### 2.6 Data collection and analysis

#### 2.6.1 Literature screening and data extraction

**Figure 1:**
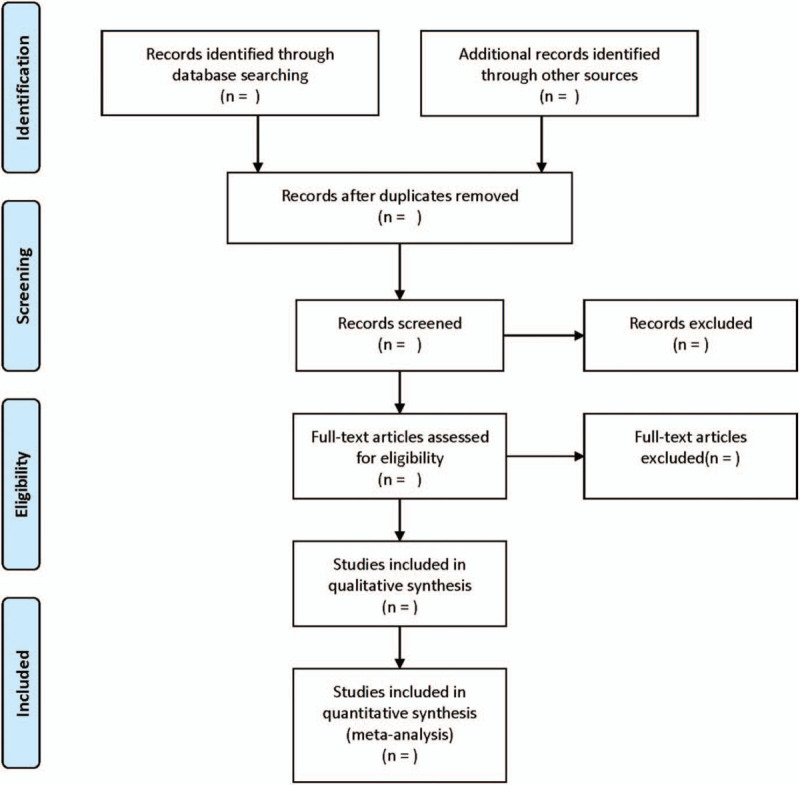
Flow diagram of study selection process.

According to the inclusion and exclusion criteria, two researchers independently completed the literature screening. By reading the full text, the data were extracted, and the final results were cross-checked. If there are different opinions, it would be further negotiated and arbitrated with a third researcher. The extraction contents are as follows: year of publication, year of publication, country, type of surgery, sample size, nerve block approach, operative site, neurolocation-based technique, drug type and dose, etc. The screening flow chart of this study is demonstrated in Figure 1.

#### 2.6.2 Assessment of risk of bias

According to the bias risk assessment tool recommended by Cochrane collaborative, two researchers evaluated literatures.

#### 2.6.3 Measures of treatment effect

The continuous variable were estimated by weighted mean difference (WMD), with 95% confidence intervals (CIs).

#### 2.6.4 Management of missing data

If any data is missing, the original data will be requested by email. If the missing data cannot be obtained, the data would be excluded from the study.

#### 2.6.5 Assessment of heterogeneity and Data synthesis

Related charts were drawn with Stata 14.0 software (Stata Corp, College Station, TX). Based on Bayesian framework, WinBUGS 1.4.3.software (MRC Biostatistics Unit, Cambridge, UK) was applied to analyze the data. Bayesian inference was carried out by adopting Markov Chain-Monte Carlo (MCMC). According to the prior probability, a posteriori probability was inferred, and the estimation and inference were conducted on the assumption that MCMC reached a stable convergence state. When running WinBUGS program, the number of iterations is set to 100,000, and the first 10,000 are used for annealing, so as to eliminate the influence of the initial value, and the simulation chain is 3. On the basis of the cumulative ranking probability map, the area size displays the probability ranking of each intervention as the best intervention. Heterogeneity test: *Q* test was preformed to qualitatively determine inter-study heterogeneity. If *P* ≥ .1, there is no inter-study heterogeneity, while if *P* < .1, there is inter-study heterogeneity. Meanwhile, *I*^2^ value was adopted to quantitatively evaluate the inter-study heterogeneity: If *I*^2^ ≤ 50%, the heterogeneity is considered to be good, and the fixed-effect model would be adopted. If *I*^2^ > 50%, it indicates significant heterogeneity, and the source of heterogeneity would be explored through subgroup analysis or sensitivity analysis. If there is no obvious clinical or methodological heterogeneity, it would be considered as statistical heterogeneity, and the random-effect model would be adopted for analysis. If there is significant clinical heterogeneity between the two groups, descriptive analysis would be carried out, while subgroup analysis is not required.

#### 2.6.6 Assessment of reporting biases

“Comparison-adjusted” funnel plot was drawn to evaluate publication bias.

#### 2.6.7 Subgroup analysis

Subgroup analysis would be conducted based on different ways of drug administration.

#### 2.6.8 Sensitivity analysis

Through the study of large weight of elimination effect, the sensitivity analysis was performed to test the stability of the results of meta-analysis.

#### 2.6.9 Grading the quality of evidence

We adopted GRADE to evaluate the quality of evidence from the following 5 aspects: risk of bias, indirectness, inconsistency, imprecision, and publication bias.^[16]^

#### 2.6.10 Ethics and dissemination

The content of this article does not involve moral approval or ethical review and would be presented in print or at relevant conferences.

## 3 Discussion

Brachial plexus block is a common anesthetic method in upper limb fracture surgery,^[17]^ with the advantages of quick effect, long maintenance time, good postoperative analgesia effect, small intervention on systemic respiration and circulation, low anesthetic cost, and definite effect.^[18–20]^ In contrast, it has been unanimously recognized by the majority of patients and surgeons. Brachial plexus block can provide good anesthetic effects, while the duration of anesthesia and analgesia is limited. The addition of adjuvant drugs to local anesthetics for peripheral nerve block is conductive to enhancing the effects of anesthesia, prolonging the duration of anesthetic analgesia and reducing side effects.^[21,22]^

Dexamethasone is a commonly used glucocorticoid, with the effects of anti-inflammation, anti-toxicity, anti-immunity, anti-shock, and so on.^[23]^ Dexamethasone has strong lipophilicity that can increase the fat solubility and pH value of the solution, facilitate the combination of the drug with the nerve sheath, and shorten the effective time.^[24]^ Some researchers add dexamethasone to local anesthetics, which can prolong the nerve block time of local anesthetics, delay the occurrence time of postoperative pain, reduce the use of opioids, and reduce the occurrence of related complications.^[25]^ Woo et al compared the effects of 2.5, 5, and 7.5 mg dexamethasone on nerve block time.^[10]^ The results proved that the nerve block time was prolonged with the increase of dexamethasone dose, but 5 mg of dexamethasone was the highest dose of medulla oblongata nerve block. Albrecht et al compared the effects of 1, 2, 3, and 4 mg dexamethasone on nerve block.^[12]^ The results indicated that there are no differences in the effects of different doses of dexamethasone on nerve block, so it was considered that the effects of dexamethasone on nerve block did not change with the change of dose. At present, there is no unified practice standard for the dose of dexamethasone in academic circles. Therefore, we adopt the method of network meta-analysis to analyze the effects of different doses of dexamethasone as a local anesthetic adjuvant on brachial plexus block to provide evidence for clinical practices.

## Author contributions

**Conceptualization:** Zhongyi Wang.

**Data curation:** Zhongyi Wang, Shuai Zhang, Meiyan Song.

**Formal analysis:** Meiyan Song.

**Funding acquisition:** Zhongyi Wang.

**Project administration:** Zhongyi Wang.

**Software:** Meiyan Song.

**Supervision:** Meiyan Song.

**Validation:** Meiyan Song, Wei An.

**Visualization:** Wei An.

**Writing – original draft:** Zhongyi Wang, Shuai Zhang.

**Writing – review & editing:** Zhongyi Wang, Shuai Zhang.
